# Case of Ruptured Aorta

**Published:** 1885-06

**Authors:** J. Paul Bush

**Affiliations:** late House Surgeon, Bristol Royal Infirmary


### CASE OF RUPTURED AORTA. By J. PAUL BUSH, late House Surgeon, Bristol Royal Infirmary.

W. H., æt. 18, a postman. The patient had been rowing, but had ceased for some considerable time, and was sitting down in the end of the boat. His companions suddenly noticed he was looking very ill; he at once lay back in the boat, and appeared to gradually sink. He was landed within a few minutes, but then appeared quite dead. This could not have been more than four or five minutes after he was first noticed to be ill. He had made no complaint whatever, and had been doing his work as a postman up to the day of his death. There was no history of rheumatism, or of any other previous illness.

*P. M. E.*—There was nothing particular to note, excepting in the heart and great bloodvessels. There was no blood whatever in the pericardial cavity. The *heart* weighed (with the portion of the aorta attached) 14 oz. The muscular walls, the valves, and orifices all appeared perfectly healthy.

A rupture through the internal and middle coats of the aorta was found. This rupture appeared to have started from a point immediately above the anterior aortic valve, which spot would correspond to the free edge of the cusp when the valve was driven back during the systole.

The rupture, starting from this point, had spread round the aorta in a slightly ascending and spiral direction, and terminated not more than a quarter of an inch higher than the level of its starting point. It had extended one complete circumference and a quarter. This rupture, in depth, extended through the elastic coats of the aorta, and had dissected the fibrous coat of the vessel to the extent of half an inch in an upward direction, and downwards this separation of the coats reached as far as the origin of the aorta from the ventricle.

There was a small amount of blood effused into this new channel. Sections of the aorta and heart substance were found, on microscopic examination, to be normal in appearance.

